# Increased Brain Activity Associated With Improved Metabolic Biomarkers: A Case Series

**DOI:** 10.1002/ccr3.71073

**Published:** 2025-10-06

**Authors:** J. A. McCallen, D. S. Oakley, G. E. Towers

**Affiliations:** ^1^ Cenegenics Medical Institute Denver Colorado USA; ^2^ WAVi Research Denver Colorado USA

**Keywords:** body fat, electroencephalogram (EEG), event related potential (ERP), hemoglobin A1C, individual peak frequency (IAF), insulin

## Abstract

This case series highlights the potential of combining dietary, exercise, and hormonal interventions to improve metabolic health markers (HbA1C, insulin, body fat) and enhance cognitive function (P300 amplitude, IAF). EEG‐based brain monitoring offers a valuable tool for tracking cognitive risk and guiding preventative strategies in aging populations.

## Graphical abstract

Bold values represent improvement. *Note: P300 V also shown as topographs of evoked voltage across the scalp (box illustrations represent regions of interest)*.

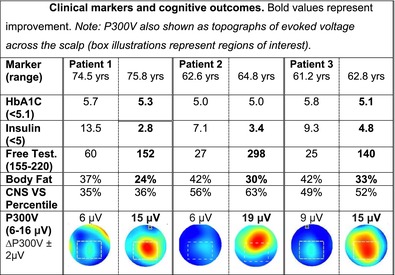

## 1 Introduction

Dementing diseases affect 5.6 million Americans and cost more than $300 billion annually [1, 2]. People of all ages are concerned about mental decline in the aging process and often cite Alzheimer's as their greatest health concern [3]. Dementia notwithstanding, general declines in mental performance that are a part of normal aging are also a concern and brain‐function optimization is a focus of a growing number of clinics. Risk factors for age‐related cognitive decline have been well studied and while this information can be used in clinic, insurance does not typically reimburse for preventive brain health services. Patients are therefore required to seek clinics offering a self‐pay model in order to receive preventive‐focused care offering health screenings that include physiologic measurements of brain activity—measurements which are otherwise not reimbursed except for neurologic diagnosis. The goal of this study is to present case studies, including patients at risk for future cognitive decline, on preventive health outcomes that include direct measures of brain function obtained alongside standard health measures.

## 2 Detailed Case Description

### 2.1 Patient Profiles and Clinical Measures

We present a case series of three male patients who are executives, under stress, who entered a clinical program for wellness and disease prevention. These patients were cognitively symptom‐free, but their cases were selected because their initial evaluation revealed elevated hemoglobin A1C and fasting insulin, low free testosterone, and excess body fat.

We focus on these markers because they have also been perceived to be important in brain health. Hemoglobin A1C (HbA1C) is a stable marker of glycation and reflects average glucose levels over time. Values at or above 5.7 are considered pre‐diabetic/diabetic and are strongly correlated with a higher incidence of vascular disease. HbA1C levels ≥ 6.5% are associated with a 2.8‐fold increased risk of dementia. HbA1C levels ≥ 7% are associated with a 5‐fold increased risk of dementia [4]. Likewise, elevated fasting serum insulin is associated with higher rates of dementia [5]. Low testosterone levels are associated with higher rates of cardiovascular mortality, obesity, insulin resistance, and dementia [6]. Finally, obesity is a primary driver of cognitive decline and can be directly measured by dual x‐ray absorptiometry as a percentage of body fat [7].

The wellness program being studied focuses on three primary interventional strategies to lower vascular disease risk and improve health:

1. *Protein‐adequate low‐glycemic diet* to reduce excess glycation and fasting insulin secretion. Dietary protein intake was increased to 150–180 g per day and dietary intake of high glycemic carbohydrates was minimized per individual to achieve a hemoglobin A1C at or below 5.1.
2. *Weightlifting and high intensity interval training* to decrease insulin resistance, and increase muscle mass, cardiovascular fitness, and cranial blood flow.
3. *Physiologic levels of testosterone supplementation* to reverse bone loss, improve muscle mass and function, reduce fat mass, and improve mood. While many myths persist about the dangers of testosterone supplementation, studies over the past 20 years have demonstrated its safety, specifically in the areas of cardiovascular and prostate health [8, 9, 10, 11, 12]. These patients were given twice weekly intramuscular testosterone cypionate to achieve a free serum testosterone level of 160‐200 pg/mL.

### 2.2 EEG/ERP Measures

Here we focus on measures relevant to age‐related cognitive decline: EEG alpha peak frequency, and an EEG event‐related potential (ERP) comprising the audio oddball P300 amplitude and latency (speed). While other EEG frequencies and other ERP metrics have also been found to be important to the task, alpha band analyses and audio P300 protocols are the most facile and accessible clinically.

The dominant frequency of the electroencephalogram (EEG) occurs in what is defined as the alpha band between 8 and 12 Hz [13, 14]. It typically is strongest occipitally in the eyes‐closed condition. The alpha band can provide information on cognitive functioning where frequency slowing, a reduction in alpha power, and a shift of strength from posterior‐to‐anterior has been observed in both normal aging and in dementia [5, 15, 16, 17, 18, 19, 20, 21, 22, 23, 24, 25, 26, 27, 28, 29, 30, 31, 32, 33, 34, 35, 36, 37]. On an individual level, an individual's peak alpha frequency (IAF) is a stable marker and can provide longitudinal patient information [38, 39, 40, 41, 42, 43, 44]. Given its high stability in the absence of neuropathological processes, IAF can provide information on neuropathological changes and can be a valuable tool to track healthy individuals for early changes, perhaps even before symptoms arise [42].

ERPs are EEG measurements time‐locked to the onset of a given stimulus. They are named according to their polarity (P for positive or N for negative) and their time of occurrence after the stimulus in milliseconds (e.g., P300). The auditory oddball protocol delivers standard repeated tones which are periodically interrupted by a different tone, or the oddball tone. This is one of the most widely studied protocols and utilizes measurements of amplitude and latency to assess the brain's cognitive ability to recognize the odd tone as different. In a healthy person this recognition occurs reliably near 300 ms post stimulus, hence the name P300. The amplitude (P300V) is thought to be proportional to the amount of attentional resources devoted to the task where the latency (P300T, the delay between stimulus delivery and recognition of the oddball tone as different) is a measure of stimulus classification speed [45]. An increase in the P300T and/or a decrease in P300V has been observed in many conditions accompanied by a decline in cognitive function and as such can be considered a non‐specific measure for aging, dementia, mild traumatic brain injury (mTBI), pre‐Alzheimer's and many others [45, 46, 47]. These metrics have also been linked to cardio health in wellness settings such as this [48].

### 2.3 Procedure

After clinical interviews and assessments, including blood samples, vascular ultrasound, VO2 max and CNS Vital Signs neurocognitive testing [49], the patients received an EEG scan with an oddball P300 using the WAVi system. Details of the scan, system, procedure, and EEG extraction has been detailed elsewhere and include a 4‐min oddball audio ERP [50, 51, 52]. The EEG study was approved by the Solutions Institutional Review Board and written informed consent was obtained from the participant before scanning.

Patients in the program are prescribed individualized treatments based upon their testing results. These patients received twice weekly intramuscular testosterone cypionate to achieve a serum free testosterone in the 75th–100th percentile for a 30–35yo man; a heavy weightlifting program 4 days weekly combined with a high intensity interval program to achieve their peak heart rate based upon VO2 Max testing; careful instruction in low‐glycemic diet sufficient to lower HbgA1C to or below 5.1%; as well as general micronutrient supplementation consisting of pharmaceutical grade multi‐vitamin/mineral, fish oil at a dose sufficient to lower hs‐CRP below 1.0 mg/L (typically 4–8 g/day) [53, 54], thyroid replacement in both T4 and T3 forms to lower TSH below 2.0mIU/L, and place freeT3 above the 50th percentile; and vitamin D optimization to a serum level of 60‐80 ng/mL [55, 56]. Patients' labs were then retested on a routine quarterly schedule, with full testing including (but not limited to) body composition, WAVi, and neurocognitive function annually.

## 3 Results

**FIGURE 1:**
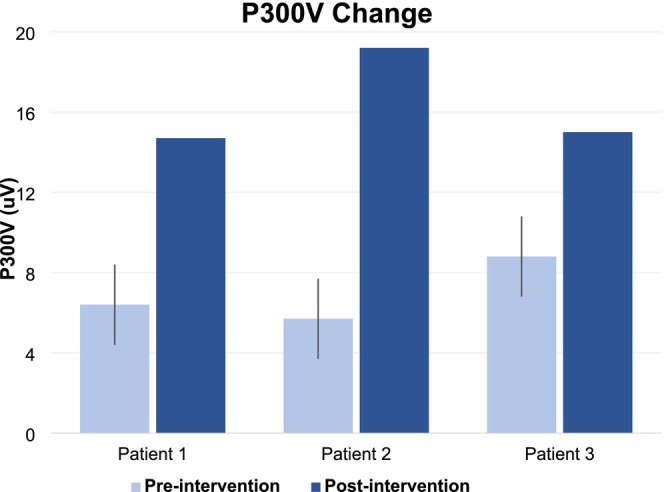
Improvement in P300 amplitudes measured pre‐ and post‐intervention. Error lines represent test–retest expectation [51].

The pre‐ and post‐treatment results for the four patients involved in this study are shown in Table 1. Changes in cognitive performance or risk are reported by changes in the average of the percentile rank of the 12 domains in the CNS test, and by changes in P300 and IAF which are compared to the expected test‐variances reported for a reference population comprising 2000 subjects as a control [51, 52]. After intervention, all of the patient's blood markers moved toward or within the clinical targets of Table 1, and all experienced a reduction in body fat. These improvements were accompanied by improved cognition. While we see marginally improved CNS scores, P300 amplitudes increased well above the normal expectation of ±2 μV (Table 1, Figure 1).

**TABLE 1:** Clinical markers and cognitive outcomes.

Marker (range)	Patient 1		Patient 2		Patient 3	
	74.5 years	75.8 years	62.6 years	64.8 years	61.2 years	62.8 years
HbA1C (< 5.1)	5.7	**5.3**	5.0	5.0	5.8	**5.1**
Insulin (< 5)	13.5	**2.8**	7.1	**3.4**	9.3	**4.8**
Free test (155–220)	60	**152**	27	**298**	25	**140**
Body fat	37%	**24%**	42%	**30%**	42%	**33%**
CNS VS percentile	35%	36%	56%	63%	49%	52%
P300V (6–16 μV), ΔP300V ± 2 μV	6 μV 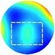	**15 μV** 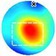	6 μV 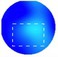	**19 μV** 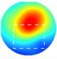	9 μV 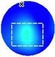	**15 μV** 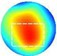

*Note:* Bold values represent improvement. P300V also shown as topographs of evoked voltage across the scalp (box illustrations represent regions of interest).

**FIGURE 2:**
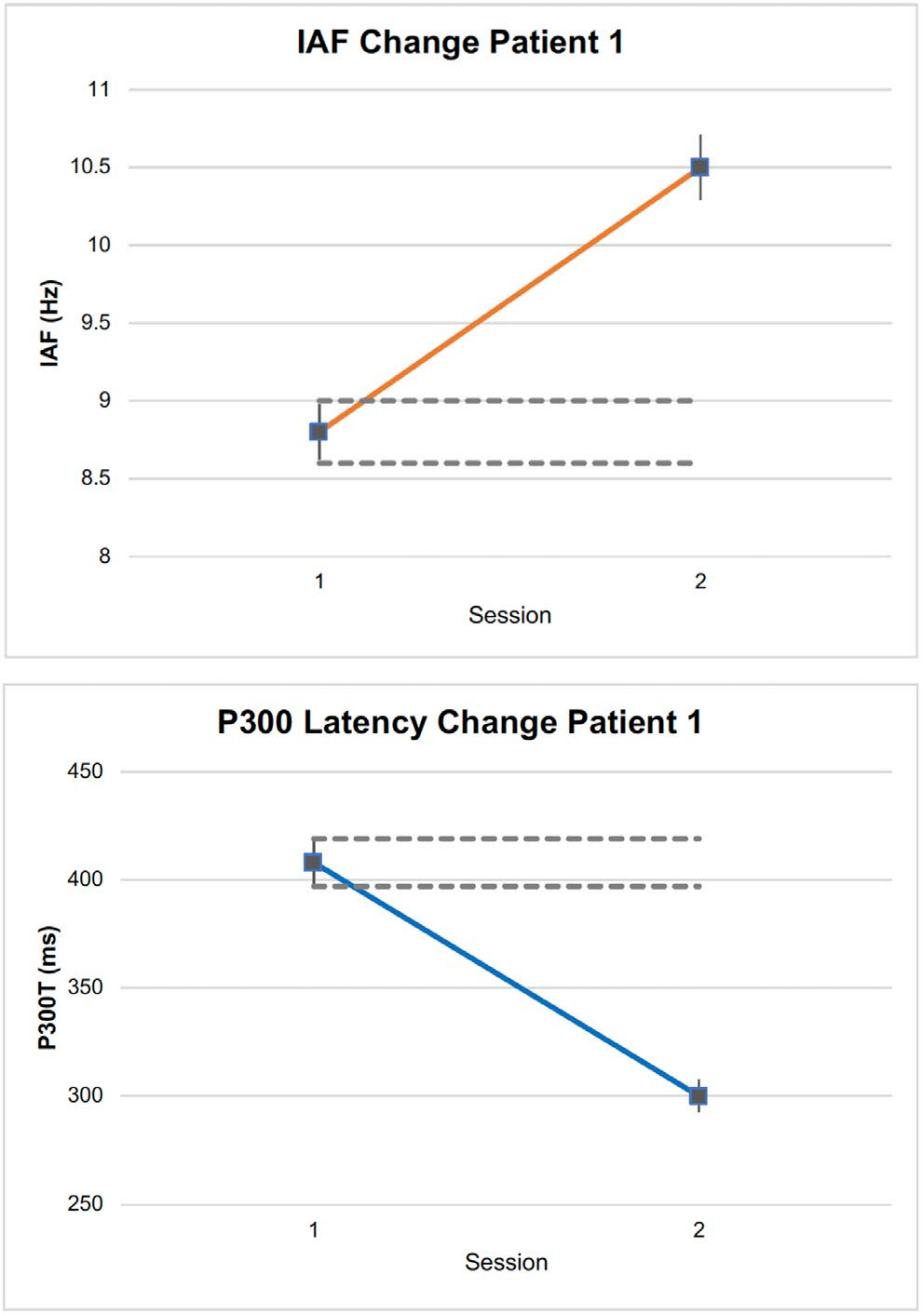
Reduction in cognitive risk markers for Patient 1. IAF increase (top graph) and a P300T decrease (bottom graph, increased speed). Dashed lines show test–retest expectation [51, 52].

None of the patients showed changes beyond expectation for the other cognitive‐decline risk markers of IAF and P300 latency, except the oldest patient (Patient 1; Figure 2) who ironically had the lowest CNS improvement. While he was symptom free and within the normal range for most CNS domains, his IAF on initial evaluation was 8.8 Hz (age‐matched target range 9.0–10.5 Hz) and his P300T was 408 ms (310–402 ms target). Changes in these trait‐based markers are early warning signs of decline that may not be apparent in neurocognitive tests as evidenced here. After intervention, his IAF sped up to a healthy 10.5 Hz and his P300T to 300 ms, both beyond expected within‐person variances (Figure 2). Alongside his 15 μV P300 amplitude, these are values expected for a typical 20‐year‐old.

## 4 Discussion

These 3 patients entered the program with no disease symptoms but had elevated A1VC, insulin, and body fat with low testosterone and low/normal evoked cognitive responses. Following treatment programs that focused on testosterone replacement, weightlifting, and low‐glycemic diet, these patients showed major improvement in most of the blood markers and body fat with an increase in cognitive resources as measured by the P300 amplitude. It is also interesting to note that the only the oldest patient had low baseline scores on other cognitive‐risk markers of P300 latency and IAF even though his CNS was normal. Following treatment these markers also showed dramatic improvement well beyond expectation to values typically seen in 20 year‐old patients.

While correlation is not causation and this case study is not designed to tease out which of the interventions, or something else, most affected cognition for each patient, these results demonstrate the need to include brain monitoring in routine evaluations. As more data are collected we can begin to answer the questions posed here, such as the importance of body fat reduction to brain longevity, an intervention that accessible to many people, versus glycemic control or less‐common interventions such as testosterone replacement. It has been known for many decades that blood pressure, for example, is a risk factor that can be modified and we now routinely measure blood pressure in otherwise symptom‐free patients in order to prescribe or test interventions. Likewise as we learn more about dementia risk factors, some of which discussed here, the measurement of brain function through oft‐reimbursable tests such as EEG/ERP can help guide prescriptions and/or track these interventions to modify these risks.

## 5 Conclusion

In these cases, we show how measures of brain function, as measured both by CNS and EEG with evoked response potentials in this instance, can be used alongside other physiological markers such as HbA1C, insulin, free testosterone, and body fat. In this case series, all patients clearly showed improved cardiometabolic health accompanied by improved cognition. In one case, the patient showed improvement in all EEG/ERP‐based cognitive‐risk markers discussed here—a cognitive risk that was not flagged by neurocognitive testing. All of these measures are readily accessible and can enable clinicians to better track preventative programs including cognition. Our goal, with this small case series, is to demonstrate that an aggressive program of hormonal, nutritional, and exercise optimization can have a powerful effect on the improvement of cognitive function with aging. We suggest that objective measures of brain function become standard in the assessment of the aging patient, long before symptoms of cognitive decline begin to appear, in order to initiate a broad range of effective strategies to reduce the incidence of cognitive decline in the general population.

## Author Contributions

**D. S. Oakley:** formal analysis, investigation, methodology, resources, software, validation, writing – original draft. **J. A. McCallen:** conceptualization, data curation, formal analysis, investigation, methodology, project administration, resources, validation, writing – original draft. **G. E. Towers:** writing – review and editing.

## Ethics Statement

This study was approved by the Solutions Institutional Review Board. Informed consent was obtained from all participants prior to their involvement in the study, and measures were taken to ensure confidentiality and privacy throughout the research process.

## Consent

Written consent for the publication of their case details and accompanying results was obtained from the patients involved in this study.

## Conflicts of Interest

David Oakley and Grace Towers are paid consultants or employees of WAVi Co. (providers of the EEG equipment). The other author declares no conflicts of interest.

## Data Availability

Access to these data can be requested by qualified researchers engaging in independent scientific research and will be provided following review and approval of a research proposal and statistical analysis plan and execution of a Data Sharing Agreement. For more information or to submit a request, please contact David Oakley, davido@wavimed.com.
